# Epigenetic Impact of Sleep Timing in Children: Novel DNA Methylation Signatures via SWAG Analysis

**DOI:** 10.3390/ijms262110615

**Published:** 2025-10-31

**Authors:** Erika Richter, Priyadarshni Patel, Yagmur Y. Ozdemir, Ukamaka V. Nnyaba, Roberto Molinari, Jeganathan R. Babu, Thangiah Geetha

**Affiliations:** 1 Department of Food, Nutrition, and Packaging Sciences, Clemson University, Clemson, SC 29631, USA; 2 Department of Nutritional Sciences, Auburn University, Auburn, AL 36849, USA; 3 Department of Environmental Health, Rollins School of Public Health, Emory University, Atlanta, GA 30322, USA; 4 Department of Mathematics & Statistics, College of Sciences and Mathematics, Auburn University, Auburn, AL 36849, USA

**Keywords:** childhood obesity, circadian rhythm, epigenetics, DNA methylation, saliva, sleep timing

## Abstract

Pediatric obesity is rising globally, and emerging evidence suggests that sleep timing may influence metabolic health through epigenetic mechanisms. This study investigated epigenome-wide DNA methylation patterns associated with bedtime in children and explored their biological relevance. Children aged 6–10 years were classified as early (≤8:30 PM) or late (>8:30 PM) bedtime groups. Saliva-derived DNA was analyzed using the Illumina Infinium MethylationEPIC BeadChip Array, and the Sparse Wrapper Algorithm (SWAG) was applied to identify differentially methylated loci. A total of 1006 CpG sites, representing 571 unique genes, were significantly associated with bedtime (*p* < 0.001). Significant methylation differences were observed between early and late bedtime groups, with *ABCG2*, *ABHD4*, *MOBKL1A*, *AK3*, *SDE2*, *PRAMEF4*, *CREM*, *CDH4*, *BRAT1*, and *SDK1* showing the most consistent variation. Functional enrichment analyses (Gene Ontology, KEGG, and DisGeNET) conducted on the SWAG-identified gene set revealed enrichment in biological processes including peptidyl-lysin demethylation, regulation of sodium ion transport, DNA repair, and lipo-protein particle assembly. Key KEGG pathways included circadian entrainment, neurotransmission (GABAergic, dopaminergic, and glutamatergic), growth hormone synthesis, and insulin secretion. DisGeNET analysis identified associations with neurodevelopmental disorders and cognitive impairment. Cross-comparison with established sleep and obesity gene sets identified ten overlapping genes(*CDH4*, *NR3C2*, *ACTG1*, *COG5*, *CAT*, *HDAC4*, *FTO*, *DOK7*, *OCLN*, and *ATXN1*). These findings suggest that variations in bedtime during childhood may epigenetically modify genes regulating circadian rhythm, metabolism, neuronal connectivity, and stress response, potentially predisposing to later-life developmental, and metabolic challenges.

## 1. Introduction

Sleep is a fundamental biological process essential for maintaining physiological, cognitive, and emotional health. In children aged 6–12 years, the American Academy of Sleep Medicine recommends 9–12 h of nightly sleep to support optimal development [1]. Insufficient sleep in this age group has been consistently associated with adverse outcomes, including daytime sleepiness, impaired cognitive performance, and increased emotional reactivity [2]. Childhood represents a critical developmental window marked by rapid growth and complex neurobiological processes, such as synaptic pruning, neural network refinement, and hormonal regulation [3], all of which are strongly influenced by sleep duration and quality.

Despite this, many children experience inadequate or disrupted sleep due to environmental and social factors. For instance, Perrault et al. (2019) demonstrated that increased evening screen exposure delays sleep onset and reduces sleep quality [4]. Similarly, Bonsu et al. (2023) found that food insecurity, disproportionately affecting low-income households, can provoke nighttime hunger and disrupt sleep [5]. In addition, Radošević-Vidaček et al. (2016) reported that parental shift work and schools operating in multiple shifts destabilize household routines and interfere with children’s sleep–wake cycles, compounding the risk of sleep insufficiency [6].

According to the National Survey of Children’s Health (NSCH, 2020–2021), 34.7% of children aged 3 to 17 years had insufficient sleep, with prevalence highest among 6–12-year-olds (37.5%) and non-Hispanic Black children (50.0%) [7]. Our previous work demonstrated that late bedtimes, independent of total sleep duration, were associated with higher BMI in elementary school-aged children [8]. Similarly, Australian studies have reported increased obesity risk among children with later bedtime and wake times [9].

Emerging evidence suggests that the relationship between sleep and health extends beyond duration to circadian alignment. Epigenetic mechanisms, particularly DNA methylation, may mediate these effects by translating behavioral patterns into lasting changes in gene expression [10,11]. DNA methylation is a key regulatory process influencing neurodevelopment, metabolism, and immune function, and it is especially dynamic during childhood, a period of heightened developmental plasticity [10]. Because the pediatric epigenome is highly sensitive to environmental and behavioral exposures, early-life sleep behaviors may exert enduring effects on gene regulation and later health outcomes [12]. Indeed, sleep disruption has been associated with altered methylation in pro-inflammatory and metabolic genes [13,14], and experimental studies demonstrate that sleep deprivation can rapidly modify methylation patterns in genes involved in synaptic plasticity [15,16]. Such alterations may represent adaptive or maladaptive programming that contributes to long-term disease susceptibility, underscoring the importance of investigating DNA methylation specifically in children [17].

While DNA methylation is the most extensively studied mechanism linking sleep and gene regulation, other epigenetic processes are also implicated. Sleep deprivation and circadian misalignment can modify histone acetylation and methylation states, particularly in genes related to synaptic plasticity and metabolism [10]. Similarly, non-coding RNAs, including microRNAs (miRNAs), respond dynamically to sleep loss, influencing pathways related to inflammation, oxidative stress, and neuronal signaling [10]. Together, these findings highlight how sleep timing and quality shape the epigenetic landscape through multiple, interrelated mechanisms. Nonetheless, DNA methylation remains a stable and quantifiable marker, making it a critical starting point for understanding how sleep behaviors become biologically embedded during childhood.

Despite mounting evidence linking sleep to epigenetic modulation, pediatric research remains scarce, with most studies conducted in adults [10]. A meta-analysis by Sammallahti et al. (2022) found no consistent associations across cohorts but identified specific CpG sites linked to sleep duration and onset [18], underscoring the need for larger, developmentally focused studies. A recent scoping review by our team further emphasized the lack of research on sleep timing, a behavioral phenotype central to circadian alignment and metabolic health [19]. While total sleep time remains a critical determinant of health, sleep timing, particularly when a child falls asleep, is increasingly recognized as a modifiable behavior influencing circadian rhythm, hormonal regulation, and metabolic outcomes [10]. Misalignment of these rhythms can have profound effects: regular patterns of eating and sleeping maintain circadian physiology, whereas recurring disruptions, such as delayed bedtimes, can impair metabolic regulation [20]. Studies indicate that circadian misalignment from delayed bedtimes disrupts lipid and glucose metabolism through alterations in core clock genes, including *CLOCK*, *BMAL1*, *CRY1*, and *PER2* [20,21].

To address this gap, the present study investigates epigenome-wide DNA methylation patterns associated with habitual sleep timing in children. By stratifying participants into early and late bedtime groups and performing high-resolution epigenome-wide analyses, we aim to identify CpG loci through which behavioral sleep patterns may exert lasting effects on neurodevelopment, metabolic function, and overall pediatric health.

## 2. Results

### 2.1. Study Participants

**Figure 1:**
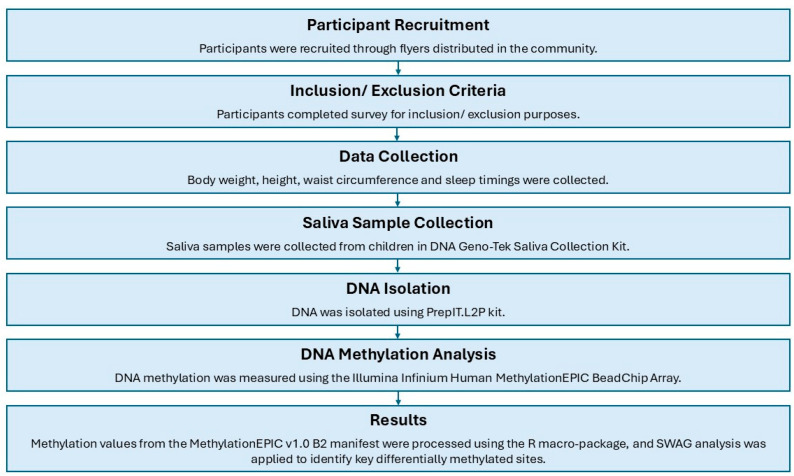
Methodology. Schematic representation of the steps involved in the analysis process.

Table 1 summarizes the demographic and anthropometric characteristics of the participants categorized into two groups based on their bedtime habits: early bedtime (before 8:30 PM) and late bedtime (after 8:31 PM). The parameters assessed include sex, age, weight, height, BMI, BMI *z*-score, WC *z*-score, and WHtR *z*-score. The mean age of the participants was 8.55 years. The obesity measurements such as BMI *z*-score, WC *z*-score, and WHtR *z*-scores were elevated in the late bedtime group (1.62 ± 0.23, 0.97 ± 0.14 and 0.75 ± 0.17, respectively) compared to the early bedtime group (0.91 ± 0.36, 0.84 ± 0.18 and 0.63 ± 0.18, respectively). However, these differences did not reach statistical significance. Overall, the trends of higher anthropometric measures were observed in children with a late bedtime compared to an early bedtime. An overview of the study methodology, including participant stratification and the analytical workflow, is illustrated in Figure 1.

### 2.2. Identification of Significantly Associated Target IDs

To identify DNA methylation differences associated with sleep timing in children, we applied the Sparse Wrapper Algorithm (SWAG) to methylation data from 865,926 CpG sites profiled using the Illumina Infinium MethylationEPIC BeadChip. This approach yielded 1006 significant target IDs, corresponding to 840 unique loci after accounting for duplicates [Table S1]. Among these, 352 target IDs exhibited hypermethylation and 488 exhibited hypomethylation in children with early bedtime compared to late bedtime (*p* < 0.001). These differentially methylated sites are mapped to 571 unique genes, detailed in Table S2. CpG sites were annotated using the array manifest to genomic features, including promoter regions, gene bodies, untranslated regions (UTRs), CpG islands, shores, shelves, and other regulatory elements [22].

**Figure 2:**
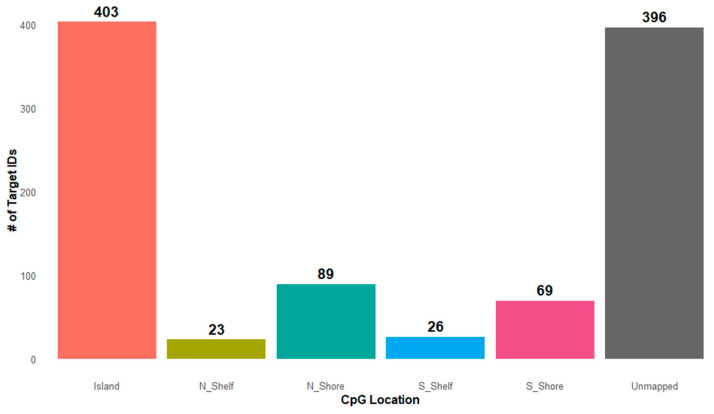
Genomic Distribution of Significant CpG Target IDs Identified by SWAG Analysis. This bar plot illustrates the distribution of 1006 significant CpG target IDs across various genomic regions. The x-axis categorizes loci based on their genomic context (e.g., island, shore, shelf, open sea), while the y-axis indicates the count of significant sites per category. Target IDs labeled as “Unmapped” refer to CpG sites lacking defined genomic coordinates. Counts are displayed above each bar to highlight the frequency of hits within each region.

Of the 610 annotated significant target ID sites, 403 (66%) were located within CpG islands, genomic regions often enriched near transcription start sites and associated with transcriptional activity (Figure 2). Detailed inspection of these island-associated CpGs revealed that 149 (37%) were within 0-200 bp of the transcription start site (TSS200), 53 (13.2%) within 200–1500 bp upstream of the TSS (TSS1500), 79 (19.6%) in the 5′UTR, 75 (18.6%) in gene bodies, 3 (0.7%) in the 3′UTR, 31 (7.7%) were intragenic, and 13 (3.2%) were categorized as unmapped regions. Examination of CpG island context further indicated that 40% of the annotated sites were located within CpG islands, with additional sites mapping to CpG shores (8.8% north, 6.9% south) and shelves (2.3% north, 2.6% south), while 39.4% could not be assigned to a defined CpG island context. Regulatory feature analysis indicated that most sites were promoter-associated (n = 240, 24%) or unclassified (n = 133, 13.3%), with a substantial fraction unmapped (n = 628, 62.7%).

Notably, several highly ranked target IDs, including cg26811976 (*PRAMEF4*), cg07891983 (*CREM*), cg04402799 (*CDH4*), and cg00136968 (*SDK1*), were categorized as unmapped by current reference annotation databases. Despite lacking specific mapping to known gene features, these loci demonstrated strong methylation differences between bedtime groups, suggesting potential regulatory relevance.

### 2.3. Top Hits of SWAG Analysis

**Figure 3:**
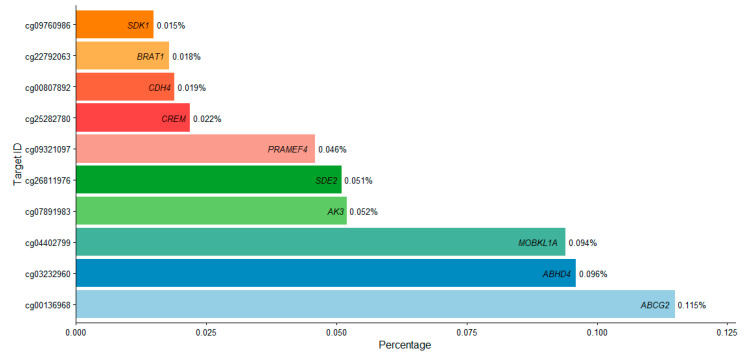
Horizontal Bar Plot of Top 10 Target IDs with Corresponding Genes by Selection Frequency in SWAG Analysis. The x-axis shows the percentage of times each target ID was selected across predictive models, while the y-axis lists the associated target IDs. Gene names are annotated on the bars in italics. Higher percentages indicate greater consistency and relevance of the target ID in distinguishing sleep timing patterns.

To deepen our understanding of the epigenetic relationship between DNA methylation and sleep timing, we focused on the top 10 target ID sites identified through SWAG analysis, selected for their statistical significance, recurrence across resampling iterations, and consistent methylation differences between early and late bedtime groups (Table 2). These target IDs were annotated to genes with established roles in neurodevelopment, transcriptional regulation, stress response, and cellular signaling. Notably, *ABCG2* (cg09760986), *ABHD4* (cg22792063), and *MOBKL1A* (cg00807892) exhibited the largest absolute methylation differences, with cg09760986 showing a Δβ of 0.115, all elevated in regard to bedtime (Figure 3). Additional targets included *AK3*, *SDE2*, *PRAMEF4*, *CREM*, *CDH4*, *BRAT1*, and *SDK1*, spanning multiple chromosomes and mapped to both CpG islands and shores (Table 2). Independent sample *t*-tests confirmed robust associations across all top sites (*p* < 0.001).

**Figure 4:**
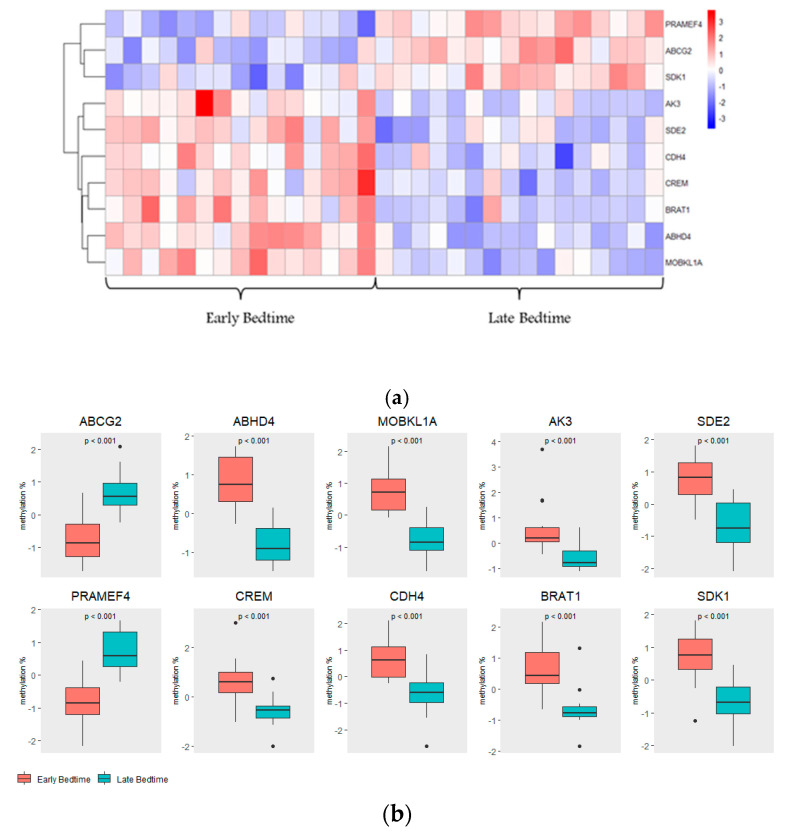
DNA methylation differences between early and late bedtime groups. (**a**) Heatmap of relative methylation (*z*-scores) for the top 10 genes across study participants. Rows correspond to the top 10 genes identified by SWAG analysis, and columns represent individual participants. Methylation levels are shown as row-wise *z*-scores, with red indicating higher and blue indicating lower relative methylation (scale: −3 to +3). Hierarchical clustering of both genes and participants highlights co-methylation patterns and grouping by bedtime category. (**b**) Boxplots of absolute methylation (β-values) for the top 10 genes by bedtime group. Boxplots display methylation levels (β-values) for early (coral) and late (teal) bedtime groups across the top 10 genes identified by SWAG analysis. The y-axis represents absolute methylation, and *p*-values from independent *t*-tests for group differences are indicated above each plot.

Heatmap visualization (Figure 4a) revealed distinct clustering of participants and genes, with methylation differences aligning with bedtime groups. Importantly, this heatmap reflects relative, standardized (*z*-score) methylation patterns across participants, which illustrate within-gene variation rather than absolute methylation direction. In contrast the boxplots in Figure 4b present absolute methylation levels (β values) for each of the top 10 genes, highlighting the true direction and magnitude of group differences. Specifically, *ABCG2* and *PRAMEF4* were hypermethylated in children with late bedtime, whereas *ABHD4*, *MOBKL1A*, *AK3*, *SDE2*, *CREM*, *CDH4*, *BRAT1*, and *SDK1* were hypomethylated in children with late bedtime compared to early bedtime. Interestingly, for *SDK1*, the *z*-score-based heatmap gives a slight reversal in trend, likely reflecting standardization effects, whereas the β value distribution clearly shows lower absolute methylation in the late-bedtime group. Together, these complementary visualizations show that sleep timing is associated with coordinated, gene-specific shifts in DNA methylation.

### 2.4. Pathway Analysis

The 571 genes identified from the SWAG analysis with *p* < 0.001 from the early versus late bedtime comparison revealed significant enrichment of multiple Gene Ontology (GO) biological processes central to epigenetic regulation and cellular function. Table 3 represents the genes involved in each of these processes. Notably, peptidyl-lysine dimethylation (*p* = 0.000078), a process involved in histone modification and transcriptional control, was highly enriched. Additional enriched pathways included amyloid precursor protein catabolism (*p* = 0.0004035), regulation of Transforming Growth Factor Beta (TGF-β) activation (*p* = 0.0007744), and positive regulation of DNA repair (*p* = 0.00109). Complementary Kyoto Encyclopedia of Genes and Genomes (KEGG) pathway analysis identified significant enrichment in pathways related to neural and synaptic function using the same list of genes (Table 4). The circadian entrainment pathway (*p* = 0.00002346) was most significantly enriched, confirming the epigenetic connection to circadian rhythm regulation. Other enriched pathways involved glutamatergic (*p* = 0.0001165), dopaminergic (*p* = 0.0001214), and GABAergic synapses (*p* = 0.0002497), underscoring the influence of sleep timing on neurotransmission and synaptic plasticity. Next, using the same set of genes, a DisGeNET disease association analysis was performed and revealed neurodevelopmental disorders as the most enriched category (*p* = 0.00003113), implicating genes including *GABRB3*, *SETD2*, *ANKRD11*, and *SCN9A* that are heavily involved in synaptic regulation, chromatin remodeling, and neuronal signaling (Table 5). Cognitive delay (*p* = 0.00003713) and mental and motor retardation (*p* = 0.00003954) were also significantly enriched phenotypes, with overlapping genes such as *SETD2*, *TBL1XR1*, *FGFR1*, and *ANKRD11* associated with synaptic plasticity, neuronal differentiation, and motor development. Together, these enriched pathways and phenotypes demonstrate that DNA methylation differences linked to sleep timing implicate critical biological processes involved in neurodevelopment, synaptic function, and genomic stability.

### 2.5. Cross-Comparison Analysis

**Figure 5:**
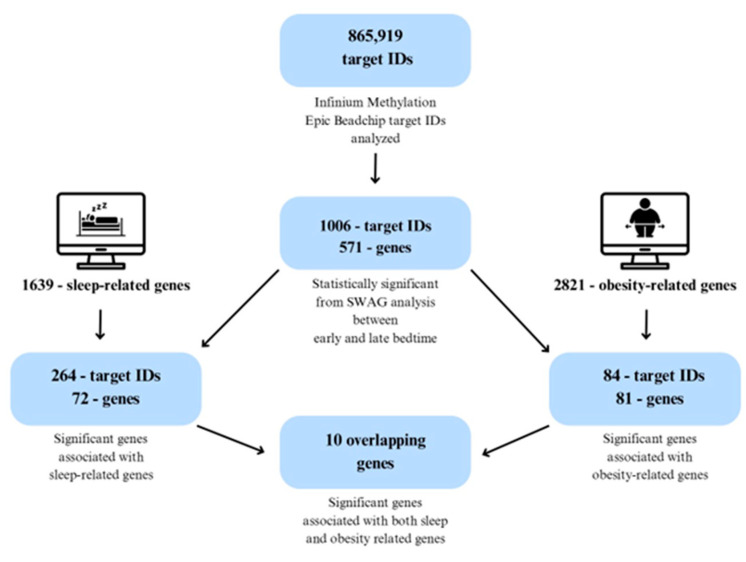
Workflow for Cross-Validation of Sleep- and Obesity-Related Genes.

**Figure 6:**
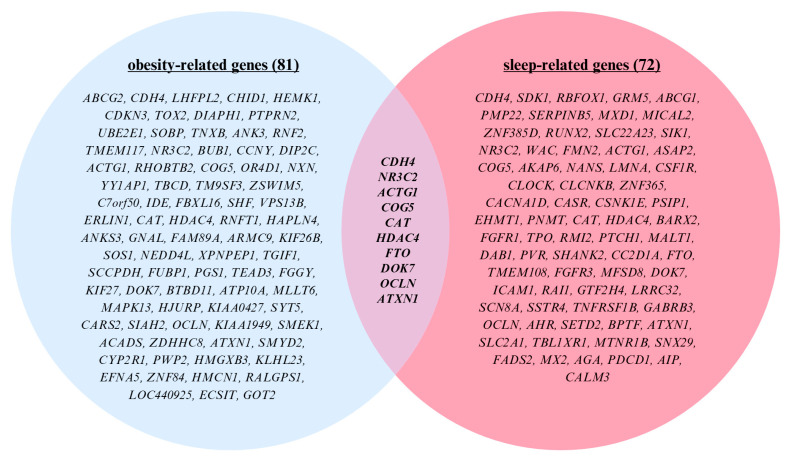
Venn Diagram Illustrating Overlap of Sleep- and Obesity-Related Genes.

Building on prior research identifying obesity-associated DNA methylation patterns in children, we examined whether the genes differentially methylated by bed timing in our study overlapped with previously established epigenetic signatures related to both sleep and obesity [23]. To contextualize our findings, we conducted an integrative cross-referencing analysis using the DisGeNET gene–disease association platform (https://www.disgenet.com/, accessed on 27 October 2025), one of the largest curated databases of gene–disease associations. From DisGeNET, we extracted two comprehensive gene sets: one associated with sleep regulation (n = 1639) and another with obesity (n = 2822). The sleep-related gene set was curated using a broad range of terms capturing diverse sleep phenotypes and disorders, including insomnia, sleep apnea (central, obstructive, and mixed types), delayed or advanced sleep phase syndromes, REM sleep behavior disorder, parasomnias, fragmented sleep, excessive daytime sleepiness, and circadian rhythm disturbances [Table S3]. These terms were selected to reflect the multifactorial nature of sleep regulation and its clinical implications. Genes were included based on established or hypothesized roles in circadian biology, neurodevelopment, respiratory regulation during sleep, and behavioral sleep disturbances. Similarly, the 2822 obesity-related genes were obtained directly from DisGeNET, using the platform’s curated list of gene-disease associations linked specifically to obesity, ensuring inclusion of genes across metabolic regulation, adipogenesis, energy balance, and inflammatory pathways relevant to obesity pathophysiology [Table S4]. We then cross-referenced these curated gene sets with our SWAG-derived list of differentially methylated genes. This analysis revealed 72 genes overlapping with sleep-related pathways and 81 with obesity-related pathways. The analytical workflow, from SWAG filtering to gene set intersection, is illustrated in Figure 5. Focusing on the intersection of these two categories, we identified 10 genes, *CDH4*, *NR3C2*, *ACTG1*, *COG5*, *CAT*, *HDAC4*, *FTO*, *DOK7*, *OCLN*, and *ATXN1* that were significantly differentially methylated between early and late bedtime groups and implicated in both sleep and obesity-related processes [Table S5]. Notably, *CDH4* emerged as a top hit in both the SWAG analysis and the set of 10 overlapping genes. These genes remained significant after applying the Benjamini-Yekutieli procedure for multiple testing correction (FDR-adjusted *p* < 0.05), as visualized in the Venn diagram in Figure 6.

## 3. Discussion

This study identified distinct DNA methylation profiles associated with children’s sleep timing, with 1006 differentially methylated CpG sites across 571 genes. Ten genes, namely *ABCG2*, *ABHD4*, *MOBKL1A*, *AK3*, *SDE2*, *PRAMEF4*, *CREM*, *CDH4*, *BRAT1*, and *SDK1*, showed the most consistent differences between early and late bedtimes (see Table A1 in Appendix A). These genes are involved in a range of biological processes, including detoxification and metabolic regulation (*ABCG2*, *ABHD4*, *AK3*), transcriptional regulation (*PRAMEF4*, *CREM*), DNA repair and genome maintenance (*SDE2*, *BRAT1*), neuronal connectivity and synaptic organization (*CDH4*, *SDK1*), and cell proliferation and developmental signaling (*MOBKL1A*), highlighting mechanistic pathways through which sleep timing may influence pediatric health.

The gene *ABCG2* is located within the South Shore of a CpG island, a region adjacent to promoters increasingly recognized for its role in modulating chromatin accessibility and tissue-specific gene expression [24]. *ABHD4*, *MOBKL1A*, *AK3*, *SDE2*, and *BRAT1* were located directly within CpG islands, regions typically associated with transcriptional regulation. Several of the other top-ranked genes, including *PRAMEF4*, *CREM*, *CDH4*, and *SDK1* were classified as “unmapped” by current annotation databases, underscoring ongoing gaps in functional epigenome annotation. Although the precise genomic coordinates of these unmapped sites remain undefined, growing evidence indicates that regulatory elements extend beyond classical promoter-associated CpG islands. Intragenic CpG sites, enhancer-associated CpGs, and other noncanonical regions can influence gene expression through mechanisms independent of classical transcriptional control [25]. The significant differences observed at unmapped sites are consistent with this evolving view, suggesting that they may represent previously unrecognized regulatory loci relevant to neurodevelopment and metabolic regulation. Variability in CpG context, from island to shore to unmapped, may help explain the diverse methylation patterns identified across bedtime groups. For example, the shore localization of ABCG2 implies a regulatory environment distinct from CpG islands, where gene expression may be particularly sensitive to environmental or behavioral exposures [24].

Functionally, *ABCG2*, which showed higher methylation in late bedtime participants, encodes an efflux transporter essential for detoxification, metabolic regulation, and circadian signaling [26]. Notably, melatonin has been shown to epigenetically regulate *ABCG2* epigenetically, suggesting that delayed bedtimes may disrupt its activity and, consequently, circadian and metabolic processes [27]. *CREM*, a transcriptional regulator of circadian rhythms and neuronal plasticity, exhibited patterns consistent with stronger circadian alignment in early sleepers [28]. *ABHD4* and *AK3*, both implicated in lipid metabolism and mitochondrial energy homeostasis, showed differential patterns that align with critical functions for neuronal health and metabolic stability [29,30].

*MOBKL1A*, a core component of the Hippo signaling pathway, exhibited differential regulation between sleep groups, implicating it in neurodevelopmental and circadian-regulated processes [31]. Genes involved in synaptic connectivity and brain structure, such as *CDH4* and *SDK1*, displayed patterns suggesting enhanced neuronal communication in early sleepers [14,32]. Late bedtime participants also demonstrated differences in genes critical for genome maintenance, including *SDE2*, essential for replication fork stability and DNA repair [33], and *BRAT1*, a mediator of DNA damage repair and cellular stress signaling [34]. *PRAMEF4*, functioning as a chromatin regulator, further illustrates how sleep timing may selectively influence gene regulatory networks [35]. Mutations in *BRAT1* have been linked to severe neurodevelopmental disorders, highlighting the importance of these pathways for brain development and genome integrity [34].

These gene-specific observations are further supported by complementary GO and KEGG pathway enrichment analyses, which highlight coherent biological themes across the broader set of 571 significant SWAG-identified genes. Enriched GO processes included peptidyl-lysin demethylation, regulation of sodium ion transport, DNA repair, and lipoprotein particle assembly, while top KEGG pathways encompassed circadian entrainment, glutamatergic and dopaminergic synapses, and aldosterone synthesis/secretion (Table 3 and Table 4). While these enrichment results do not directly indicate methylation directionality, they underscore the potential functional impact of sleep timing-associated epigenetic variation and highlight new avenues for mechanistic investigation.

Cross-comparison analyses revealed ten additional genes, *CDH4*, *NR3C2*, *ACTG1*, *COG5*, *CAT*, *HDAC4*, *FTO*, *DOK7*, *OCLN*, and *ATXN1*, that were differentially methylated by sleep timing and have also been implicated in obesity-related pathways (Table A2 in Appendix A provides full abbreviations and functions), suggesting an epigenetic interface connecting circadian regulation and metabolic health. For instance, Yin et al. (2023) emphasized the role of *FTO* as a well-established obesity-associated gene, noting that its variants influence fat storage, energy balance, and metabolic disease risk through both genetic and epigenetic mechanisms, including RNA modification pathways [36]. In a complementary line of research, Kong et al. (2018) demonstrated that *HDAC4* promotes neuroprotection and angiogenesis via epigenetic regulation of signaling cascades such as HIF-1α–VEGF and CREB–BDNF, suggesting relevance for stress adaptation and sleep-related neuroplasticity [37].Extending this epigenetic framework to stress regulation and behavior, Quing et al. (2021) explored mechanisms by which *NR3C2*, encoding the mineralocorticoid receptor, may influence cortisol responses, mood, and cognitive outcomes through epigenetic regulation [38,39,40].

Beyond these, other identified genes support complementary pathways connecting sleep and metabolism. *CDH4* mediates cell adhesion and neurodevelopment, potentially affecting circadian regulation [14]. *ACTG1* encodes a cytoskeletal protein critical for neuronal plasticity and metabolic function [41]. *COG5* participates in Golgi apparatus function and protein glycosylation, processes vital to circadian protein stability and metabolic enzyme activity [42]. *CAT* encodes catalase, an antioxidant enzyme that mitigates oxidative stress, a known contributor to sleep disruption and metabolic dysregulation [43]. *DOK7* is involved in neuromuscular junction formation and intracellular signaling, potentially influencing energy metabolism with circadian pathways [44]. *OCLN* maintains blood–brain barrier integrity, with implications for neuroinflammation and sleep disorders [45]. *ATXN1*, a transcriptional regulator, may mediate epigenetic connections between circadian disruption, metabolic function and neurological outcomes [46].

Collectively, these findings strengthen the evidence linking sleep timing to obesity through shared epigenetic pathways. The overlapping genes identified here converge on biological processes central to energy balance, cellular signaling, and neurodevelopment, providing mechanistic support for the observed association between sleep timing and metabolic risk. Consistent with this, Chawla et al. (2025) identified delayed sleep schedules as a key contributor to adolescent obesity, independent of diet or physical activity [47]. Our results extend this work by suggesting that differences in sleep timing may influence the methylation of genes involved in circadian regulation and metabolic homeostasis. Importantly, prior studies indicate that improving sleep behaviors can reverse such methylation alterations, underscoring the potential reversibility of these epigenetic effects and the modifiable nature of sleep as a determinant of long-term metabolic health [48,49].

To our knowledge, this study represents the first analysis of the identified genes (*ABCG2*, *ABHD4*, *MOBKL1A*, *AK3*, *SDE2*, *PRAMEF4*, *CREM*, *CDH4*, *BRAT1*, *SDK1*, *NR3C2*, *ACTG1*, *COG5*, *CAT*, *HDAC4*, *FTO*, *DOK7*, *OCLN*, and *ATXN1*) in the context of sleep and obesity-related DNA methylation changes in children. Among these, *FTO* is the only gene previously implicated in pediatric populations, having surfaced in studies linking sleep and childhood obesity [19]. This highlights the first comprehensive epigenetic analysis of these genes, providing insights into molecular mediators of sleep timing and its impact on pediatric health.

This study has several limitations. The relatively small sample size may have reduced statistical power to detect modest effects. Sleep timing was assessed using parent-reported questionnaires, which are vulnerable to recall bias and may not fully reflect children’s actual sleep behaviors. Although wake-up times were generally constrained by school schedules, total sleep duration was not directly assessed, and other aspects of sleep quality were not evaluated, which may have influenced the findings. Potential confounding environmental factors, including evening light exposure, screen use, and household routines, were also not comprehensively measured. Despite these constraints, the findings provide preliminary yet compelling evidence that habitual sleep timing in childhood may shape the epigenome during a sensitive developmental window.

This study focused primarily on sleep timing because our lab’s research found that 71% of children with late bedtimes were obese, compared to only 29% of children with early bedtimes [8]. Yet, sleep duration represents another important dimension that could influence epigenetic patterns. Future analyses could compare children sleeping less than 8.5 h versus those exceeding 8.5 h to evaluate dose-dependent effects on DNA methylation [9]. However, our findings suggest that timing itself, independent of total duration, may play a unique role in shaping the pediatric epigenome. Environmental factors such as evening light exposure, screen use, and electronic devices use may modulate sleep behaviors and should be considered in future studies [4]. Incorporating these variables will help disentangle the relative contributions of sleep timing versus duration and contextual factors in pediatric epigenetic regulation, while reinforcing the importance of prioritizing sleep timing as a modifiable behavioral target.

Overall, these findings underscore the importance of integrating epigenetic perspectives into pediatric sleep research to elucidate the biological mechanisms linking lifestyle behaviors to health outcomes. The sleep timing-focused behavioral interventions may modulate epigenetic outcomes and reduce the development or risk of obesity and related metabolic disorders. Future studies should aim to validate these findings in larger, longitudinal cohorts and investigate the reversibility of sleep-associated methylation changes through lifestyle modifications and targeted interventions.

## 4. Materials and Methods

### 4.1. Participants

Children aged 6 to 10 years were recruited from Lee County and Macon County, Alabama. Parents expressing interest in the study contacted the research team via email or phone. A preliminary phone screening was conducted to confirm eligibility and exclude children with medical conditions such as diabetes, cardiovascular disease, or sleep apnea, as well as those taking medications or antibiotics during the study period. Written informed consent was obtained from parents or legal guardians, and assent was obtained from the children. Demographic information was collected, including date of birth, sex, race/ethnicity, weekday bedtime, maternal education level, and family income. Saliva samples were collected, and a schematic overview of the study design is presented in Figure 1. 

### 4.2. Anthropometric Measurements

Anthropometric data were collected following the World Health Organization (WHO) guidelines. Height and weight were measured without shoes and with light clothing. Height was recorded to the nearest 1/8 inch using a stadiometer, and weight to the nearest 4 ounces using a digital scale (WB-800H plus; Tanita Corporation, Tokyo, Japan). BMI was calculated using the Centers for Disease Control and Prevention (CDC) growth charts. BMI *z*-scores (adjusted for age and sex) were derived based on WHO 2007 growth reference data [50]. Waist circumference was measured to assess fat distribution, using a measuring tape positioned midway between the lowest ribs and the iliac crest, to the nearest 0.1 cm. Additionally, the waist/height ratio (WHtR) was calculated based on the LMS tables from NHANES III developed by Sharma et al. [51]. All calculations were performed in R Studio version 4.5.1. (Posit Software, Boston, MA, USA).

### 4.3. Isolation of Salivary DNA

Saliva was collected using the Oragene Geno-Tek saliva collection kit (Catalog #OGR-500; Ottawa, ON, Canada) during participants’ visits. Collections were generally conducted in the after-school period, between 3 and 5 pm on weekdays, and participants were required to abstain from food for at least one hour prior to collection to minimize potential dietary influences. Following the manufacturer’s instructions, samples were incubated at 50 °C for three hours. DNA was isolated from a 500 µL aliquot using the PrepIT.L2P DNA isolation kit (Catalog #PT-L2P-5; DNAgenotek, Ottawa, ON, Canada). Each sample was labeled and stored at −20 °C. DNA concentration was measured using a NanoDrop ND-1000 spectrophotometer (Thermo Fisher Scientific, Inc., Wilmington, DE, USA). Samples were sent to the University of Minnesota Genomics Center for genome-wide methylation profiling using the Illumina Infinium MethylationEPIC BeadChip, which interrogates over 860,000 methylation sites.

### 4.4. Sodium Bisulfite Conversion and Infinium Arrays

DNA samples (250–750 ng) were treated with sodium bisulfite using the EZ DNA Methylation Kit (Zymo Research, Irvine, CA, USA). Following the manufacturer’s protocol, bisulfite-treated DNA underwent amplification, fragmentation, purification, and hybridization to the Illumina Human MethylationEPIC BeadChip. Arrays were cleaned and scanned using the Illumina HiScan System. IDAT data were processed with Illumina’s Genome Studio software (V2011.1) using the MethylationEPIC v-1-0 B2 manifest. Annotations provided by the array manifest were used to assign each CpG site to genomic features, including promoter regions, gene bodies, untranslated regions, CpG islands and adjacent regions, and other regulatory elements. Background normalization was performed using negative control probes to generate methylation β-values, for subsequent analyses.

### 4.5. Data Analysis

Signal intensities and methylation levels were extracted using Genome Studio by Illumina. Probes with data from two beads or fewer or with detection *p*-values greater than 0.01 were excluded. To obtain methylation β levels, signal intensities were standardized, and noise was eliminated using negative control probes. β-values were calculated as the ratio of methylation probe intensity to total intensity.

Statistical analysis was conducted using the R statistical software. After quality control, 865,926 target IDs were retained for each participant. SWAG [52], a heuristic model selection approach, was used to identify differentially methylated loci associated with early versus late bedtimes. Unlike single-model approaches, SWAG selects multiple sparse models with few variables, improving interpretability and stability [53,54,55,56] and aligning with the “Predictability, Computability, Stability” (PCS) framework [57]. This approach has been successfully applied in diverse biomedical contexts, including cancer diagnosis, melanoma classification, COVID-19 severity prediction, and ADHD detection [58,59,60,61]. Sparse modeling was essential due to the small sample size, which limits reliable estimation of many effects jointly. A detailed description of SWAG is given in Supplementary Materials.

Within SWAG, logistic regression predicted sleep pattern classification from target IDs and phenotypic variables. Model quality was evaluated using Akaike Information Criterion (AIC). Post-processing of the models selected by SWAG (see statistical appendix), identified 4210 models (2487 with two variables each and the remainder with three variables each). Variable importance was determined from the frequency of each screened variable across the SWAG collection (library) of models, identifying those most strongly associated with sleep patterns. Considering the exploratory nature of this study, independent *t*-tests (without family-wise error corrections) supported the reliability of the findings, with all 10 most frequent target IDs showing significant differences (*p* < 0.05) between early and late bedtime. Additionally, 1639 sleep-related and 2821 obesity-related genes were selected for functional enrichment analysis using the DisGeNET platform (https://www.disgenet.org, accessed on 4 October 2024).

## 5. Conclusions

In conclusion, variations in bedtime during childhood may epigenetically alter genes governing circadian regulation, metabolism, neuronal connectivity, and stress responses, thereby increasing long-term risk for developmental, cognitive, and metabolic challenges. These results highlight the translational significance of integrating epigenetic perspectives into pediatric sleep research and emphasize the value of early, sleep-focused behavioral interventions to promote a healthy epigenetic landscape and optimize long-term pediatric health outcomes.

## Figures and Tables

**Table 1:** General Characteristics of the study population.

Parameter	Total	Early Bedtime (Before 8:30 pm)	Late Bedtime (After 8:31 pm)	*p*-Value
Total Participants	31	15	16	-
Sex (male/female)	17/14	9/6	8/8	-
Age (years)	8.55 ± 0.24	8.22 ± 0.39	8.86 ± 0.29	0.201
Weight (kg)	36.05 ± 2.27	33.58 ± 3.58	38.36 ± 2.84	0.305
Height (cm)	134.35 ± 2.23	132.24 ± 3.36	136.33 ± 2.97	0.369
BMI (kg/m^2^)	19.42 ± 0.64	18.51 ± 0.98	20.28 ± 0.81	0.174
BMI *z*-score	1.27 ± 022	0.91 ± 0.36	1.62 ± 0.23	0.111
WC *z*-score	0.91 ± 0.11	0.84 ± 0.18	0.97 ± 0.14	0.556
WHtR *z*-score	0.69 ± 0.12	0.63 ± 0.18	0.75 ± 0.17	0.637

Values are presented as mean ± standard deviation. BMI = Body Mass Index; WC = Waist Circumference; WHtR = Waist-to-Height Ratio.

**Table 2:** Top 10 Target IDs with Corresponding Genes associated with Sleep Timing from SWAG Analysis.

No.	Target ID	GENE	CHR	LOCATION
1	cg09760986	*ABCG2*	4	S_Shore
2	cg22792063	*ABHD4*	14	Island
3	cg00807892	*MOBKL1A*	4	Island
4	cg25282780	*AK3*	9	Island
5	cg09321097	*SDE2*	1	Island
6	cg26811976	*PRAMEF4*	1	~
7	cg07891983	*CREM*	10	~
8	cg04402799	*CDH4*	20	~
9	cg03232960	*BRAT1*	7	Island
10	cg00136968	*SDK1*	7	~

CHR = Chromosome; S_Shore = South Shore; Island = CpG Island; ~ = representing loci with unmapped or unspecified genomic positions from the Infinium MethylationEpic Manifest database.

**Table 3:** Top 10 GO (Gene Ontology) Processes that were enriched due to Early vs. Late bed timings.

Index	Name	*p*-Value	Genes
1	Peptidyl-Lysine Dimethylation (GO:0018027)	7.4 × 10^−5^	*SETD2*, *SETD7*, *SMYD2*, *EHMT1*
2	Amyloid Precursor Protein Catabolic Process (GO:0042987)	0.00038	*ADAM19*, *APH1B*, *ADAM10*, *ABCG1*
3	Regulation Of Sodium Ion Transport (GO:0002028)	0.00042	*NKAIN1*, *NEDD4L*, *SIK1*, *ATP1A1*, *ANK3*, *FGF12*
4	Regulation Of Transforming Growth Factor Beta Activation (GO:1901388)	0.00074	*TNXB*, *LRRC32*, *LTBP1*
5	Positive Regulation of DNA Repair (GO:0045739)	0.00099	*PRKCG*, *SMARCE1*, *TMEM161A*, *EYA2*, *DPF1*, *RUVBL1*, *RPS3*, *FMN2*, *SMARCA4*
6	Positive Regulation of Cardiac Muscle Hypertrophy (GO:0010613)	0.00116	*TRPC3*, *HAND2*, *AKAP6*, *PRKCA*
7	Peptidyl-Lysine Monomethylation (GO:0018026)	0.00117	*SETD7*, *SMYD2*, *EHMT1*
8	High-Density Lipoprotein Particle Assembly (GO:0034380)	0.00171	*ZDHHC8*, *PRKACA*, *PRKACB*
9	Positive Regulation of Lipase Activity (GO:0060193)	0.00171	*PDPK1*, *FGFR3*, *FGFR1*
10	Positive Regulation of Protein Sumoylation (GO:0033235)	0.00171	*HDAC4*, *PIAS3*, *RWDD3*

**Table 4:** Top 10 KEGG (Kyoto Encyclopedia of Genes and Genomes) pathways that were enriched due to methylation changes between Early bedtime vs. Late bed timings.

Index	Name	*p*-Value	Genes
1	Circadian entrainment	0.00002046	*PRKCG*, *KCNJ5*, *CREB1*, *MTNR1B*, *ADCY3*, *PRKCA*, *CACNA1D*, *CALM3*, *PRKACA*, *PRKACB*, *CACNA1H*, *GNG13*
2	Glutamatergic synapse	0.0001022	*PRKCG*, *GRM5*, *HOMER2*, *ADCY3*, *HOMER3*, *PRKCA*, *CACNA1D*, *PRKACA*, *SLC1A6*, *PRKACB*, *SHANK2*, *GNG13*
3	Dopaminergic synapse	0.0001058	*PRKCG*, *KCNJ5*, *PRKCA*, *CACNA1D*, *PPP2R5C*, *GNG13*, *MAPK1*, *3 GNAL*, *CREB1*, *CALM3*, *PRKACA*, *PRKACB*, *CLOCK*
4	Aldosterone synthesis and secretion	0.0001107	*PRKCG*, *KCNJ5*, *CREB1*, *ADCY3*, *PRKCA*, *CACNA1D*, *CALM3*, *ATP1A1*, *PRKACA*, *PRKACB*, *CACNA1H*
5	GABAergic synapse	0.0002234	*GABRB3*, *PRKCG*, *SLC12A5*, *GABRA5*, *ADCY3*, *PRKCA*, *CACNA1D*, *PRKACA*, *PRKACB*, *GNG13*
6	Retrograde endocannabinoid signaling	0.0003312	*PRKCG*, *GABRB3*, *KCNJ5*, *NDUFA12*, *GABRA5*, *ADCY3*, *PRKCA*, *CACNA1D*, *GNG13*, *MAPK13*, *GRM5*, *PRKACA*, *PRKACB*
7	Aldosterone-regulated sodium reabsorption	0.0005771	*PRKCG*, *PDPK1*, *NEDD4L*, *PRKCA*, *ATP1A1*, *NR3C2*
8	Growth hormone synthesis, secretion and action	0.0006086	*MAP2K3*, *PRKCG*, *CREB1*, *IGFBP3*, *ADCY3*, *PRKCA*, *CACNA1D*, *PRKACA*, *SOS1*, *PRKACB*, *MAPK13*
9	Insulin secretion	0.0007717	*PRKCG*, *CREB*, *SLC2A1*, *ADCY3*, *PRKCA*, *CACNA1D*, *ATP1A1*, *PRKACA*, *PRKACB*
10	Hedgehog signaling pathway	0.001028	*EVC*, *KIF3A*, *PTCH1*, *CSNK1E*, *PRKACA*, *PRKACB*, *GLI3*

**Table 5:** Top 10 DisGeNET results that were enriched due to Early vs. Late bed timings.

Index	Name	*p*-Value	Genes
1	Neurodevelopmental Disorders	0.00003113	*GABRB3*, *RBFOX1*, *SETD2*, *ANKRD11*, *EHMT1*, *VPS13B*, *ANK3*, *SSTR4*, *RNF2*, *SMARCA4*, *RHOBTB2*, *PARD3B*, *RAI1*, *APH1B*, *TBL1XR1*, *SCN8A*, *WAC*, *SHANK2*
2	Cognitive delay	0.00003713	*GABRB3*, *SETD2*, *NUP107*, *NDUFA12*, *SLC2A1*, *EHMT1*, *FMN2*, *PEPD*, *SOBP*, *ACTG1*, *MFSD8*, *PMPCA*, *ACADS*, *MFSD2A*, *CEP135*, *HYMAI*, *ARMC9*, *VPS13B*, *PCCA*, *TBL1XR1*, *SCN8A*, *SIK1*, *CARS2*, *FTO*, *HDAC4*, *ANKRD11*, *BRAT1*, *NEDD4L*, *CACNA1D*, *RAI1*, *TPO*, *DPH1*, *LMNA*, *SLC13A5*, *RREB1*, *SEC23B*, *BUB1*, *SMARCE1*, *SLC12A5*, *SLC35A3*, *PTCH1*, *TBCD*, *SMARCA4*, *OCLN*, *OGDH*, *NF1*, *TAF6*, *FGFR3*, *CC2D1A*, *FGFR1*
3	Mental and motor retardation	0.00003954	*GABRB3*, *SETD2*, *NUP107*, *NDUFA12*, *SLC2A1*, *EHMT1*, *FMN2*, *PEPD*, *SOBP*, *ACTG1*, *MFSD8*, *PMPCA*, *ACADS*, *MFSD2A*, *CEP135*, *HYMAI*, *ARMC9*, *VPS13B*, *PCCA*, *TBL1XR1*, *SCN8A*, *SIK1*, *CARS2*, *FTO*, *HDAC4*, *ANKRD11*, *BRAT1*, *NEDD4L*, *CACNA1D*, *RAI1*, *TPO*, *DPH1*, *LMNA*, *SLC13A5*, *RREB1*, *SEC23B*, *BUB1*, *BPTF*, *SMARCE1*, *SLC12A5*, *SLC35A3*, *PTCH1*, *TBCD*, *SMARCA4*, *DIAPH1*, *OCLN*, *OGDH*, *NF1*, *TAF6*, *FGFR3*, *CC2D1A*, *FGFR1*
4	Small midface; Decreased projection of midface; Hypotrophic midface; Midface retrusion	0.00004885	*HDAC4*, *SF3B4*, *NXN*, *PTCH1*, *AGL*, *EHMT1*, *RUNX2*, *RAI1*, *TBL1XR1*, *LMNA*, *NF1*, *WAC*, *SOS*, *FGFR3*, *FGFR1*
5	Small head	0.0001059	*SF3B4*, *FTO*, *HDAC4*, *RBM28*, *NUP107*, *ANKRD11*, *BRAT1*, *SLC2A1*, *EHMT1*, *ACTG1*, *RAP1A*, *AGA*, *KDSR*, *SLC13A5*, *NANS*, *BUB1*, *MFSD2A*, *SMARCE1*, *ENTPD1*, *CEP135*, *SLC35A3*, *VPS13B*, *SMARCA4*, *TUFM*, *DIAPH1*, *OCLN*, *TBL1XR1*, *SCN8A*, *NF1*, *TAF6*, *B9D2*, *FGFR3*, *FGFR1*
6	Decreased circulating renin level	0.0001204	*KCNJ5*, *CYP11B1*, *CACNA1D*, *NR3C2*
7	Triangular head shape; Wedge shaped head	0.0002024	*DPH1*, *PTCH1*, *GLI3*, *ACTG1*, *FGFR1*
8	Global developmental delay	0.0002664	*GABRB3*, *SETD2*, *NUP107*, *NDUFA12*, *SLC2A1*, *EHMT1*, *FMN2*, *PEPD*, *SOBP*, *ACTG1*, *MFSD8*, *PMPCA*, *ACADS*, *MFSD2A*, *CEP135*, *HYMAI*, *ARMC9*, *VPS13B*, *PCCA*, *TBL1XR1*, *SCN8A*, *CAT*, *SIK1*, *CARS2*, *FTO*, *HDAC4*, *ANKRD11*, *BRAT1*, *NEDD4L*, *CACNA1D*, *RAI1*, *TPO*, *DPH1*, *LMNA*, *SLC13A5*, *RREB1*, *SEC23B*, *BUB1*, *SMARCE1*, *SLC12A5*, *SLC35A3*, *PTCH1*, *TBCD*, *SMARCA4*, *OCLN*, *OGDH*, *NF1*, *PMP22*, *TAF6*, *FGFR3*, *CC2D1A*, *FGFR1*
9	Triglycerides measurement	0.0002834	*FTO*, *ABCC3*, *STARD13*, *DNAH17*, *PINX1*, *VPS13B*, *FMN2*, *AKR1C4*, *PEPD*, *INHBC*, *HAPLN4*, *TMEM241*, *AFF1*, *NR3C2*, *SUGCT*, *FADS2*, *RAP1A*, *TMEM117*, *DOK7*, *PSMD1*, *CLOCK*, *ABCG1*
10	Broad face	0.0003828	*RAI1*, *TBL1XR1*, *PTCH1*, *AGA*

## Data Availability

All data generated or analyzed during this study are included in this published article [and its Supplementary Information files].

## Supplementary Materials

The following supporting information can be downloaded at https://www.mdpi.com/article/10.3390/ijms262110615/s1. References [62,63,64,65,66] is cited in the Supplementary Materials.

## Abbreviations

The following abbreviations are used in this manuscript:

ABCG2	ATP-binding cassette sub-family G member 2
ABHD4	Abhydrolase domain containing 4
ACTG1	Actin gamma 1
ADHD	Attention-deficit/hyperactivity disorder
AIC	Akaike Information Criterion
AK3	Adenylate kinase 3
ANKRD11	Ankyrin repeat domain-containing protein 11
ATXN1	Ataxin 1
BMI	Body mass index
BMAL1	Brain and muscle ARNT-like 1 (circadian clock gene, also ARNTL)
BRAT1	BRCA1-associated ATM activator 1
BY	Benjamini–Yekutieli (False Discovery Rate control method)
CAT	Catalase
CDC	Centers for Disease Control and Prevention
CDH4	Cadherin 4
CI	Confidence Interval
CLOCK	Circadian locomotor output cycles kaput (core circadian gene)
COG5	Component of oligomeric Golgi complex 5
CREM	cAMP responsive element modulator
CpG	Cytosine-phosphate-Guanine dinucleotide
CRP	C-reactive protein
CRY1	Cryptochrome circadian regulator 1
DNA	Deoxyribonucleic acid
DOK7	Docking protein 7
EWAS	Epigenome-wide association study
FDR	False Discovery Rate
FGFR1	Fibroblast growth factor receptor 1
FTO	Fat mass and obesity-associated gene
GABA	Gamma-aminobutyric acid
GABRB3	Gamma-aminobutyric acid receptor subunit beta-3
GO	Gene Ontology
HDAC4	Histone deacetylase 4
IDAT	Intensity Data File (Illumina)
IL-6	Interleukin-6
IRB	Institutional Review Board
KEGG	Kyoto Encyclopedia of Genes and Genomes
LMS	Lambda-Mu-Sigma method (for growth references)
MOBKL1A	MOB kinase activator-like 1A
NHANES	National Health and Nutrition Examination Survey
NR3C2	Nuclear receptor subfamily 3 group C member 2
NSCH	National Survey of Children’s Health
OCLN	Occludin
OR	Odds Ratio
PCS	Predictability, Computability, Stability framework
PER2	Period circadian regulator 2
PRAMEF4	Preferentially expressed antigen in melanoma family member 4
REM	Rapid eye movement
RNA	Ribonucleic acid
SCN9A	Sodium voltage-gated channel alpha subunit 9
SDK1	Sidekick cell adhesion molecule 1
SDE2	Silencing-defective 2 homolog
SE	Standard Error
SETD2	SET domain containing 2 (lysine methyltransferase)
SWAG	Sparse Wrapper Algorithm
TBL1XR1	Transducin beta-like 1 X-linked receptor 1
TGF-β	Transforming Growth Factor Beta
UTR	Untranslated region
WHO	World Health Organization
WHtR	Waist-to-height ratio
β	Beta coefficient (regression estimate)

## Appendix A

**Table A1:** Top 10 Genes Identified from SWAG analysis and Their Functions.

No.	Gene	Full Name/Protein	Key Function	Length (aa)	Methylation Direction (Late Bedtime)
1	*ABCG2*	ATP-binding cassette sub-family G member 2	Broad substrate transporter; exports porphyrins, heme, sphingosine-1-P, urate, and xenobiotics; contributes to detoxification and homeostasis	655	Hyper
2	*ABHD4*	Abhydrolase domain-containing protein 4	Lysophospholipase; hydrolyzes N-acyl phosphatidylethanolamines to generate precursors for endocannabinoids (e.g., anandamide)	342	Hypo
3	*MOBKL1A*	MOB kinase activator 1A	Activator in Hippo signaling; regulates organ size, cell proliferation, and apoptosis via LATS1/2–YAP1 pathway	221	Hypo
4	*AK3*	Adenylate kinase 3, mitochondrial	Maintains nucleotide homeostasis; catalyzes GTP:AMP and ITP:AMP phosphotransferase reactions	227	Hypo
5	*SDE2*	Stress response regulator SDE2	DNA replication and cell cycle control; binds PCNA to modulate translesion DNA synthesis and DNA damage responses	451	Hypo
6	*PRAMEF4*	PRAME family member 4	Member of PRAME gene family; putative role in transcriptional regulation and oncogenesis	478	Hyper
7	*CREM*	cAMP response element modulator	Transcription factor binding CRE sites; functions as activator or repressor; essential for spermatogenesis	300	Hypo
8	*CDH4*	Cadherin-4 (R-cadherin)	Calcium-dependent adhesion protein; mediates homophilic cell–cell adhesion; important in retinal development	916	Hypo
9	*BRAT1*	BRCA1-associated ATM activator 1	Regulates DNA damage response and mitochondrial function; stabilizes mTOR pathway proteins	821	Hyper
10	*SDK1*	Sidekick cell adhesion molecule 1	Large adhesion molecule; mediates lamina-specific synaptic connections in retina via homophilic interactions	2213	Hypo

Hyper = hypermethylation, Hypo = hypomethylation. Functional relationships among these genes were further explored using the STRING database (https://string-db.org/, accessed on 28 October 2025) to generate protein–protein interaction networks.

**Table A2:** Relevance and Function of the 10 Overlapping Genes.

No.	Gene	Full Name/Protein	Key Function	Length (aa)	Methylation Direction (Late Bedtime)
1	*CDH4*	Cadherin-4 (R-cadherin)	Calcium-dependent adhesion protein; mediates homophilic cell–cell adhesion; important in retinal development	916	Hypo
2	*NR3C2*	Mineralocorticoid receptor (MR)	Nuclear receptor for aldosterone and cortisol; regulates ion/water balance, blood pressure, and electrolyte homeostasis	984	Hyper
3	*ACTG1*	Actin, cytoplasmic 2	Ubiquitous cytoskeletal protein; essential for cell motility, structure, and intracellular transport	375	Hypo
4	*COG5*	Conserved oligomeric Golgi subunit 5	Component of the COG complex; required for normal Golgi trafficking and glycoprotein processing	860	Hyper
5	*CAT*	Catalase	Antioxidant enzyme; degrades hydrogen peroxide, protecting cells from oxidative stress; supports immune cell growth	527	Hypo
6	*HDAC4*	Histone deacetylase 4	Epigenetic regulator; deacetylates histones, repressing transcription; roles in development, muscle maturation, and cancer pathways	1084	Hyper
7	*FTO*	Fat mass and obesity-associated protein (RNA demethylase)	Demethylates N6-methyladenosine (m6A) in RNA; regulates gene expression, energy balance, adipogenesis, and obesity risk	505	Hyper
8	*DOK7*	Docking protein 7	Activates MUSK receptor; essential for neuromuscular junction formation and acetylcholine receptor clustering	504	Hypo
9	*OCLN*	Occludin	Integral tight junction protein; regulates paracellular permeability and barrier integrity	522	Hypo
10	*ATXN1*	Ataxin-1	Chromatin-binding protein; represses Notch signaling, regulates RNA metabolism; implicated in brain development and neurodegeneration	815	Hyper

Hyper = hypermethylation, Hypo = hypomethylation. Functional relationships among these genes were further explored using the STRING database (https://string-db.org/, accessed on 28 October 2025) to generate protein–protein interaction networks.
