# Supplementary material for: Epigenetic Impact of Sleep Timing in Children: Novel DNA Methylation Signatures via SWAG Analysis
Source: Int J Mol Sci. 2025 Oct 31;26(21):10615. doi: 10.3390/ijms262110615 (PMC12607692; doi:10.3390/ijms262110615)
Supplement: Supplementary file 1 [file ijms-26-10615-s001.zip › Supplementary Statistical Appendix_proof.pdf]

Statistical analysis was performed using the R open-source statistical software, and it can be found in the following link: https://github.com/yagmuryavuzozdemir/Sleep_study. The dataset is comprised of $n = 31$ participants with 865,926 corresponding target IDs obtained from the participants' saliva samples and identified from the Illumina Human MethylationEPIC BeadChip method. The primary objective of the study is to identify variations in methylation patterns associated with early or late sleep timings in children, considering also a few phenotypic variables consisting of the anthropometric measurements on the participants. The response variable is binary, indicating whether participants go to bed early or late based on the sleep parameters set for the study.

Based on the goal of the analysis, we need an approach that allows us to find the most important variables (i.e., target IDs and phenotypic variables) that distinguish sleep timing patterns in children. This is commonly called a high-dimensional problem in the field of Data Science since there are many possible explanatory/predictive variables (almost 866,000 in our case), but much fewer observations (31 participants in our case). Many screening procedures can potentially be used to extract the few variables that can predict sleeping patterns, but they tend to overlook collinearities in the data as well as potential interaction effects. Ideally, we would like to fit a classifier to the data that also selects the most important variables (e.g., target IDs): a standard solution is a penalized regression such as Lasso; however, this method does not tend to work very well in these particularly high-dimensional problems (and small sample sizes). Other classifiers could be used, such as Support Vector Machines or Random Forests, but these generally work better on larger sample sizes, and, in this case, there are not enough observations to detect patterns across all variables. The best solution would therefore be to run a simple and interpretable model due to the data size, but this is not possible due to the high number of variables. Moreover, the small number of observations can lead to spurious discoveries (e.g., non-significant variables that are evaluated as statistically significant) when considering a single model.

For all the above reasons, we choose to use a recently developed technique consisting of a novel heuristic model selection method called the Sparse Wrapper Algorithm (SWAG) [1]. This fits into a new paradigm in Data Science which addresses the so-called Rashomon Effect: the existence of multiple models which have almost-equivalent predictive accuracy for a given problem and dataset. The advantages of this new paradigm are various, among which stability of variable importance metrics [2]. Since we want to identify a few important variables (target IDs) for future studies, we want to find collections of good sparse models (i.e., models with few variables), and SWAG is tailored to this problem. This algorithm can make use of any modelling technique to find, select, and connect important variables through a collection of good models when there are many possible variables to evaluate (i.e., high-dimensional problems). These advantages have led to successful applications of SWAG and its previous version in areas such as the diagnosis of leukemia [3], bowel cancer [4], melanoma [5], COVID-19 intensive care treatment [6], breast cancer detection [7], and the diagnosis of ADHD from brain images [8]. Further applications are discussed in [1], highlighting various advantages of SWAG, including achieving comparable accuracy with a smaller number of variables. Moreover, recently proposed approaches put forward similar ways of finding sparse sets of models (instead of a single one) to stabilize interpretations [9]. In addition, the "Predictability, computability, and stability" (PCS) framework proposed by Yu

and Kumbier (2020) requires exploring the stability of predictions by considering many alternative analytic perturbations, which this kind of approach directly addresses [10].

For this dataset, as said earlier, using simpler and interpretable models is recommended, given that the number of observations is not enough to accurately detect more complex relationships in the data (using semi- or non-parametric approaches such as Decision Trees or Generalized Additive Models). Therefore, we chose to use simple logistic regression, which, aside from having a more stable structure to separate sleep patterns, also has interpretable coefficients that describe how each variable contributes to the presence of a certain sleep pattern or not. Since logistic regression cannot be run in high-dimensional problems and since we want to obtain more stable variable importance metrics, we run logistic regression within the SWAG, which we briefly describe below (a more detailed and precise description of the algorithm can be found in [1]).

The overall aim of the SWAG is to identify a collection of good models that include up to a limited number of variables: we call this number $\mathrm{p}_{\max}$. The latter is chosen by the user based on their needs or, in our case, to ensure that enough observations are available to reasonably estimate the impact of all the variables considered in the model. For this purpose, we use the Event Per Variable criterion, which generally states that the number of observations per class (i.e., per sleep pattern state) must be at least four times larger than the number of variables in the model [11, 12]. This rule brings us to determine $p_{max} = 4$, which means that the SWAG will only build and evaluate models with 4 variables or fewer. This, however, does not mean that only 4 variables will be evaluated in the algorithm. The algorithm has a first screening step where, if we define p as being the total number of variables (i.e., target IDs and phenotypic variables), then the SWAG estimates p models (which we can denote with M) each with one variable and ranks them based on their prediction error (we call this D). For this analysis, since we are using logistic regression, we chose to use the Akaike Information Criterion (AIC) as the prediction error metric [13]. Once this is done, the user defines a percentile (proportion) that we call α , which specifies how many of the best models (and therefore variables) will be used to build models in the following steps of the algorithm. These will be the screened variables, and, in our case, we choose $\alpha = 0.001$ to focus only on the 0.1% of the best models, resulting in the selection of 871 variables in this first screening step. Since these variables did not include the phenotypic variables, these were added to the screened variables so that they are given the possibility of being evaluated in the following steps of the SWAG. We denote the screened variables as $\tilde{X}$. Since it is relatively simple to estimate models with only one variable, even with missing observations (therefore avoiding possible small biases in estimation due to imputation), it becomes more problematic when building larger models: we therefore imputed missing observations in the screened variables using kNN (k-Nearest Neighbors), where the parameter k, i.e. number of neighboring observations, was chosen to be 6 based on the common rule $the\ k \propto \sqrt{n}$ [14].

In the following steps, i.e., general step, the SWAG builds m models for each dimension $2 \leq d \leq p_{max}$, where d represents the number of variables included in each model and m represents the maximum number of distinct models (i.e., models with different combinations of variables included in them) to be evaluated for each dimension d. Therefore, for $d = 2$ the SWAG samples m, distinct combinations of variable pairs from the screened variables $\tilde{X}$ and ranked based on their

prediction error D. The best α percent of these models is saved (into $\widetilde{M}_2$) which are then used as a basis to build models in dimension $d = 3$ by sampling them and combining them with distinct variables sampled from the screened variables $\tilde{X}$. Having built m distinct models, each with 3 variables in them, the best α percent of these models are saved into $\widetilde{M}_3$, which, as in the previous step, are combined with distinct screened variables to build m distinct models with 4 variables (of which the best α percent of these models are saved into $\widetilde{M}_4$). Once we have the best α models for each dimension $1 \le d \le p_{max} = 4$, the user can perform a post-processing step, which, in our case, consisted of finding the dimension d for which the median prediction error is lowest. For this dimension, we take the δ-quantile of the prediction error (AIC in our case) and use this as the maximum error we admit in the SWAG models. This implies that we only keep all models, for all dimensions, that have a prediction error below this value. As a result of this, the total number of models selected after post-processing is 4210, of which 2487 are 2-dimensional (i.e., contain two variables each) and the rest are 3-dimensional (i.e., contain three variables each), implying that 4-dimensional models were not performing as well as the lower-dimensional ones.

A summary representation of the SWAG is shown in the flowchart below.

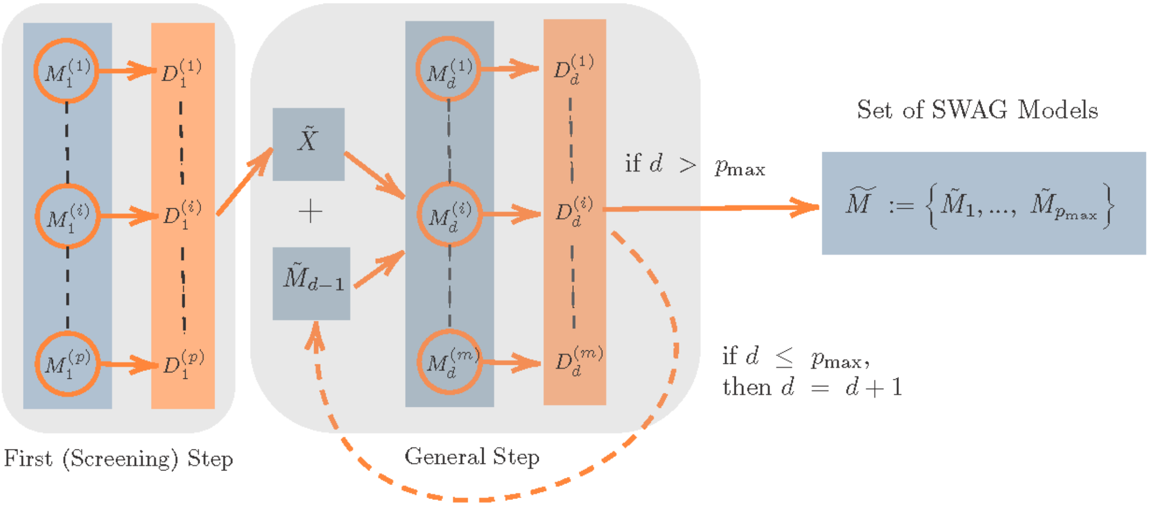

Flowchart Overview of SWAG

Since our goal is to extract important variables that are related to the sleep patterns of children, we concentrated on the variable importance metric produced by SWAG. To do so, we analyze the collection of models (library) selected by SWAG. Since each model is built on a different variables, for each screened variable, say X_i, the number of models containing X_i is counted to define the "frequency" of a variable. The frequency table of the 10 most selected variables can be found in Table S1. To support the reliability of our findings and given the exploratory nature of the study, we conducted several independent Student t-tests for each variable (without family-wise error rate correction) to assess differences in the methylation status of significant target IDs between early

and late sleepers. At a 0.05 significance level, all 10 t-tests yielded significant results, indicating that the most frequent 10 variables also differ between the two groups of children in their sleeping patterns.

References

1. Molinari, R., Bakalli, G., Guerrier, S., Miglioli, C., Orso, S., Karemera, M., & Scaillet, O. (2020). Swag: A wrapper method for sparse learning. arXiv preprint arXiv:2006.12837.
2. Fisher, A., Rudin, C., & Dominici, F. (2019). All models are wrong, but many are useful: Learning a variable's importance by studying an entire class of prediction models simultaneously. Journal of Machine Learning Research, 20(177), 1–81.
3. Guerrier, S., Mili, N., Molinari, R., Orso, S., Avella-Medina, M., & Ma, Y. (2016). A predictive-based regression algorithm for gene network selection. Frontiers in Genetics, 7, 97. https://doi.org/10.3389/fgene.2016.00097
4. Mili, N., Molinari, R., Ma, Y., & Guerrier, S. (2016). Differentiating inflammatory bowel diseases by using genomic data: Dimension of the problem and network organization. Human Genomics, 10. https://doi.org/10.1186/s40246-016-0070-9
5. Branca, M., Orso, S., Molinari, R. C., Xu, H., Guerrier, S., Zhang, Y., & Mili, N. (2018). Is nonmetastatic cutaneous melanoma predictable through genomic biomarkers? Melanoma Research, 28(1), 21–29. https://doi.org/10.1097/CMR.0000000000000414
6. Parisi, N., Janier-Dubry, A., Ponzetto, E., Pavlopoulos, C., Bakalli, G., Molinari, R., Guerrier, S., & Mili, N. (2020). Non-applicability of validated predictive models for intensive care admission and death of COVID-19 patients in a secondary care hospital in Belgium. medRxiv. https://doi.org/10.1101/2020.11.15.20231567
7. Miglioli, C., Bakalli, G., Orso, S., Karemera, M., Molinari, R., Guerrier, S., & Mili, N. (2022). Evidence of antagonistic predictive effects of miRNAs in breast cancer cohorts through data-driven networks. Scientific Reports, 12(1), 5166. https://doi.org/10.1038/s41598-022-09143-4
8. Ozdemir, Y. Y., Nukala, N. C. P., Molinari, R., & Deshpande, G. (2024). A multi-model framework to explore ADHD diagnosis from neuroimaging data. Journal of Data Science, 22(2).
9. Kissel, N., & Mentch, L. (2024). Forward stability and model path selection. *Statistics and Computing, 34*(82). https://doi.org/10.1007/s11222-024-10395-8
10. Yu, B., & Kumbier, K. (2020). Veridical data science. *Proceedings of the National Academy of Sciences, 117*(8), 3920–3929. https://doi.org/10.1073/pnas.1901326117
11. Moons, K. G. M., de Groot, J. A. H., Bouwmeester, W., Vergouwe, Y., Mallett, S., Altman, D. G., Collins, G. S., & Reitsma, J. B. (2014). Critical appraisal and data extraction for systematic reviews of prediction modelling studies: The CHARMS checklist. *PLoS Medicine, 11*(10), e1001744. https://doi.org/10.1371/journal.pmed.1001744
12. Pavlou, M., Ambler, G., Seaman, S. R., De Iorio, M., & Omar, R. Z. (2016). Review and evaluation of penalised regression methods for risk prediction in low-dimensional data

with few events. *Statistics in Medicine, 35*(7), 1159–1177. https://doi.org/10.1002/sim.6792
13. Akaike, H. (1974). A new look at the statistical model identification. IEEE Transactions on Automatic Control, 19(6), 716–723.
14. Cover, T., & Hart, P. (1967). Nearest neighbor pattern classification. IEEE Transactions on Information Theory, 13(1), 21–27.
